# Comparison of hybrid coronary revascularization and minimally invasive multivessel CABG for multivessel coronary artery disease

**DOI:** 10.3389/fcvm.2026.1887132

**Published:** 2026-09-29

**Authors:** Ting Ren, Haoyi Yao, Zhaohui Cheng, Zibo Lin, Jiapei Qiu, Qiang Zhao

**Affiliations:** 1 Department of Cardiovascular Surgery, Ruijin Hospital Affiliated to Shanghai Jiao Tong University School of Medicine, Shanghai, China; 2 Department of Cardiovascular Surgery, Beijing Anzhen Hospital, Capital Medical University, Beijing, China

**Keywords:** CABG, complication, graft patency, HCR, MICS-CABG

## Abstract

**Background:**

This study aimed to compare the perioperative and early-to-mid-term outcomes of hybrid coronary revascularization (HCR) and minimally invasive multivessel coronary artery bypass grafting (MICS-CABG) for treating multivessel coronary artery disease (MVCAD).

**Methods:**

We conducted a retrospective observational cohort study including MVCAD patients who underwent HCR or MICS-CABG at Ruijin Hospital between January 2019 and December 2024. The primary endpoint was the incidence of major perioperative complications within 30 days after surgery, including all-cause mortality, myocardial infarction (MI), and stroke. Secondary endpoints included 1-year all-cause survival and 1-year graft or target-vessel patency. Propensity score matching (PSM) was performed to reduce baseline imbalance between the two groups. Kaplan–Meier analyses were used to evaluate 1-year survival.

**Results:**

A total of 169 patients were included, of whom 91 underwent HCR and 78 underwent MICS-CABG. After 1:1 PSM, 132 patients remained, with 66 patients in each group. Major perioperative complications within 30 days after surgery were comparable. In the HCR group, MI occurred in 1 patient (1.52% vs. 0.00%; *P* = 1.000), stroke in 2 patients (3.03% vs. 0.00%; *P* = 0.476), and death in 1 patient (1.52% vs. 0.00%; *P* = 1.000), whereas no such events occurred in the MICS-CABG group. The MICS-CABG group had significantly longer operative time (250 vs. 160 min, *P* < 0.001) and greater 24-hour postoperative drainage (420 vs. 185 mL, *P* < 0.001). Postoperative respiratory complications were numerically more frequent after MICS-CABG. The 1-year survival rate was comparable between the HCR and MICS-CABG groups after PSM (98.5% vs. 98.4%; log-rank *P* = 0.996). The 1-year left anterior descending artery (LAD) revascularization patency rate was comparable between the HCR and MICS-CABG groups (94.6% vs. 90.0%; *P* = 0.366), as was non-LAD target-vessel patency (86.5% vs. 87.0%; *P* = 0.922).

**Conclusions:**

HCR and MICS-CABG provided comparable perioperative safety, early-to-mid-term survival, and 1-year revascularization patency in selected patients with MVCAD. Treatment strategy may be individualized according to coronary lesion characteristics and cardiopulmonary function.

## Introduction

Coronary artery bypass grafting (CABG) remains a standard treatment for complex multivessel coronary artery disease (MVCAD). However, conventional CABG requires a median sternotomy, which is associated with considerable surgical trauma and reduced patient acceptance (1). Consequently, the development of minimally invasive coronary surgery has received growing attention (2). For patients with MVCAD, two representative minimally invasive approaches have emerged: minimally invasive multivessel coronary artery bypass grafting (MICS-CABG) and hybrid coronary revascularization (HCR). HCR combines the long-term patency advantage of a left internal mammary artery (LIMA) graft to the left anterior descending artery (LAD) with the minimally invasive benefits of percutaneous coronary intervention (PCI) for non-LAD lesions (3). Unlike HCR, MICS-CABG aims to achieve complete multivessel revascularization entirely through a small thoracic incision, without planned PCI. Theoretically, MICS-CABG preserves the advantage of complete surgical revascularization while reducing the trauma associated with conventional sternotomy. However, the procedure is technically demanding due to limited operative space and requires high-level surgical expertise and close team coordination (4). Although previous studies have suggested that HCR or MICS-CABG may provide perioperative safety and mid- to long-term outcomes comparable to conventional CABG, evidence directly comparing HCR with MICS-CABG remains scarce (4–6). Therefore, this study aimed to compare the perioperative safety, early- to mid-term survival, and 1-year revascularization patency of HCR and MICS-CABG in patients with MVCAD.

## Methods

### Study population

This single-center retrospective cohort study screened consecutive patients with MVCAD who underwent HCR or MICS-CABG at Ruijin Hospital between January 2019 and December 2024. Patients were eligible if they underwent first-time surgical revascularization and had complete clinical data. Patients requiring concomitant cardiac procedures, such as transcatheter aortic valve replacement or atrial fibrillation ablation, were excluded.

Patients were categorized into either the HCR or MICS-CABG group. HCR was defined as a staged hybrid strategy combining minimally invasive surgical grafting and PCI, with the interval between surgery and PCI ≤60 days. The sequence of treatment could be PCI first, commonly in patients with acute coronary syndrome(ACS), or surgery first. MICS-CABG was defined as complete multivessel revascularization performed exclusively through minimally invasive surgery, without planned or actual PCI during the index treatment period.

### Surgical procedures

#### MICS-CABG group

Patients were placed supine under general anesthesia with double-lumen endotracheal intubation to allow right-lung ventilation. The left hemithorax was elevated approximately 30°. The LIMA was harvested under direct vision using a dedicated minimally invasive retractor. The right internal mammary artery (RIMA) and additional conduits, including the saphenous vein graft (SVG) or radial artery (RA), were harvested when clinically indicated according to the planned revascularization strategy.

All distal anastomoses were performed on the beating heart with the aid of a cardiac stabilizer. The LIMA was anastomosed to the LAD, and additional grafts were placed as indicated to the diagonal branches, left circumflex territory, posterolateral left ventricular branches, posterior descending artery, or right coronary artery. Intraoperative graft assessment was routinely performed after completion of the anastomoses.

Postoperative dual antiplatelet therapy, consisting of aspirin 100 mg once daily plus either ticagrelor 90 mg twice daily or clopidogrel 75 mg once daily, was initiated on postoperative day 1 unless contraindicated.

#### HCR group

LIMA harvesting was performed similarly, with a shorter 5–8 cm incision. After systemic heparinization, the pericardium was opened and suspended. A cardiac stabilizer facilitated end-to-side LIMA-to-LAD anastomosis. Postoperative antiplatelet therapy was administered according to the same regimen used in the MICS-CABG group, with PCI for non-LAD lesions typically performed on days 4–5.

### Patient data collection and follow-up

Clinical data, including demographics, comorbidities, surgical details, perioperative events, and postoperative outcomes, were collected from electronic medical records and operative reports. Follow-up was conducted via outpatient visits and telephone interviews.

The primary endpoint was 30-day major perioperative complications, including all-cause mortality, myocardial infarction (MI), and stroke. Secondary endpoints included overall survival during follow-up and 1-year graft or target-vessel patency, re-exploration for bleeding, respiratory complications, new-onset atrial fibrillation, wound-related complications, duration of mechanical ventilation, ICU stay, and postoperative hospital stay. At 1 year postoperatively (12 ± 3 months), LAD graft patency and non-LAD target-vessel patency were evaluated using coronary computed tomography angiography (CCTA) or coronary angiography (CAG). LAD patency analysis was restricted to patients with an LAD bypass graft. For non-LAD territories, patency assessment included bypass grafts in the MICS-CABG group and coronary stents after PCI in the HCR group. Target-vessel patency was graded according to the FitzGibbon classification. Grade A was defined as a patent vessel without stenosis or with less than 50% stenosis; grade B was defined as a patent vessel with greater than 50% stenosis; and grade O was defined as complete occlusion. In the present study, both grade A and grade B were considered to indicate patency (7).

The study protocol was approved by the Institutional Review Board of Ruijin Hospital(Approval letter No. 2025-202) and conducted in accordance with the Declaration of Helsinki and institutional guidelines. Written informed consent was obtained from all participants or their legal representatives.

### Statistical analysis

Continuous variables are presented as mean ± standard deviation(SD) or median with interquartile range, as appropriate. Categorical variables are expressed as frequencies and percentages. Between-group comparisons were performed using the Student t test or Mann–Whitney U test for continuous variables and the *χ*^2^ test or Fisher exact test for categorical variables, as appropriate.

Propensity scores were calculated using multivariable logistic regression including age, gender, ACS, number of diseased vessels, and left main coronary artery disease. Patients were matched 1:1 using nearest-neighbor matching without replacement and a caliper width of 0.2 SD of the logit of the propensity score. Covariate balance before and after matching was assessed using standardized mean differences, with values less than 0.10 considered indicative of adequate balance (Supplementary Figure 1).

Overall survival was estimated using the Kaplan–Meier method, and differences between groups were assessed with the log-rank test. All tests were two-sided, and a *P* value less than 0.05 was considered statistically significant. Analyses were performed using R software, version 4.3.3, and IBM SPSS Statistics, version 26.0.

## Results

### Patient selection and baseline characteristics

Between January 2019 and December 2024, 171 patients underwent HCR or MICS-CABG at the study center. Two patients were excluded because of missing key clinical data; both were patients in the HCR group who underwent staged PCI at local hospitals, for whom detailed procedural information was unavailable. Accordingly, 169 patients were included in the final analysis, including 91 in the HCR group and 78 in the MICS-CABG group. After 1:1 propensity score matching(PSM), 132 patients remained, with 66 patients in each group. The patient selection and grouping process is shown in Figure 1.

**Figure 1:**
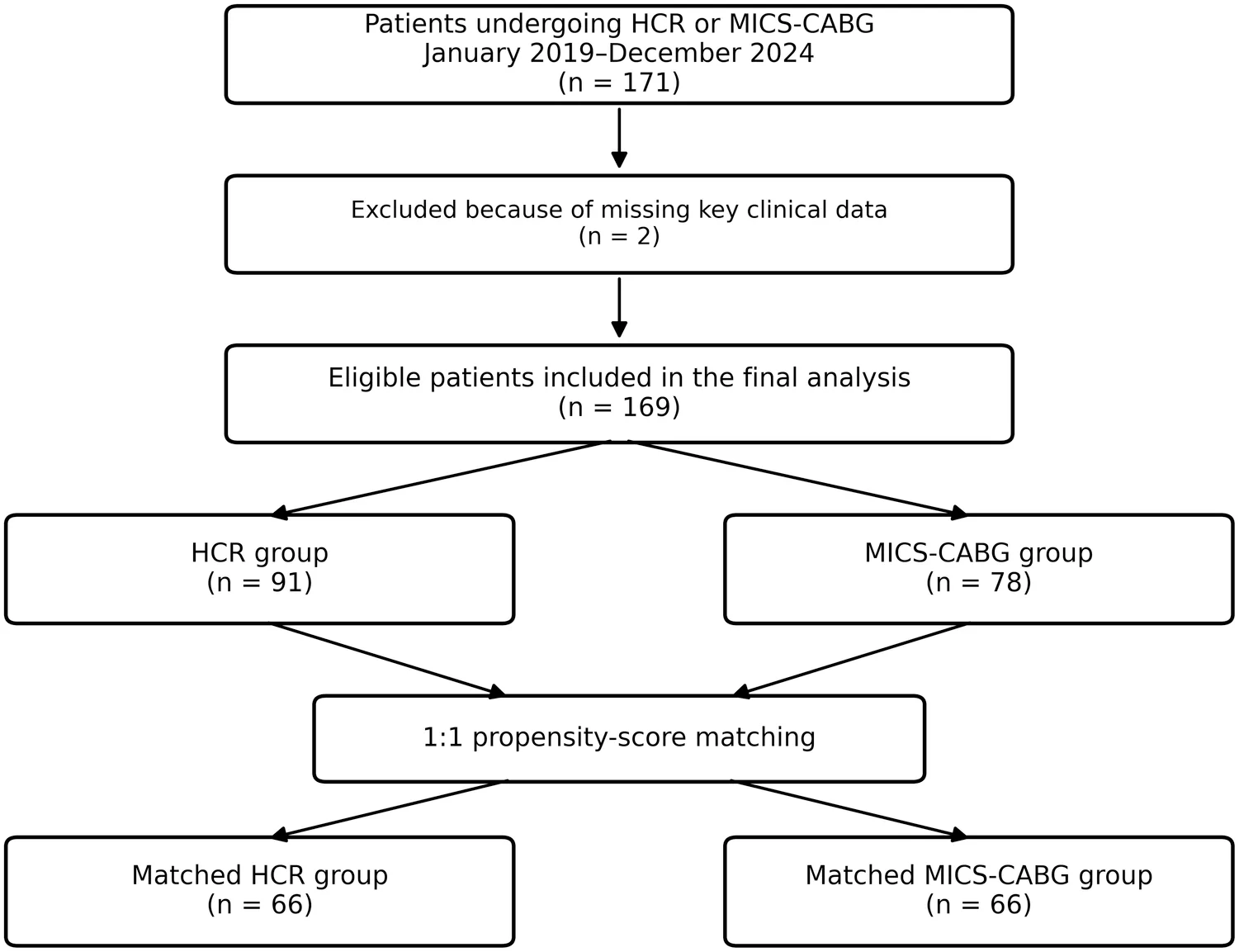
Flow chart of patient selection.

Baseline characteristics are summarized in Table 1. Both groups were generally comparable regarding demographics, comorbidities, and cardiac function. Before matching, ACS was more frequent in the HCR group than in the MICS-CABG group (71.4% vs. 53.8%, *P* = 0.018). The HCR group tended to have more patients with Canadian Cardiovascular Society(CCS) class III to IV angina (82.4% vs. 70.5%, *P* = 0.067), three-vessel disease (81.3% vs. 69.2%, *P* = 0.068), and left main coronary artery disease (36.3% vs. 23.1%, *P* = 0.063). By contrast, renal insufficiency (23.1% vs. 12.1%, *P* = 0.059), chronic obstructive pulmonary disease(COPD) (15.4% vs. 6.6%, *P* = 0.065), and cerebrovascular disease (34.6% vs. 22.0%, *P* = 0.068) were more frequent in the MICS-CABG group.

**Table 1:** Baseline characteristics of patients undergoing HCR and MICS-CABG.

Variable	Total (*n* = 169)	HCR (*n* = 91)	MICS-CABG (*n* = 78)	*P* Value
Age (years), median (Q1, Q3)	69.00 (62.00, 73.00)	69.00 (63.00, 75.00)	67.00 (62.00, 71.75)	0.085
Male, *n* (%)	133 (78.7)	72 (79.1)	61 (78.2)	0.885
BMI (kg/m^2^), median (Q1, Q3)	24.24 (22.48, 25.86)	24.45 (22.86, 25.95)	23.93 (22.23, 25.78)	0.599
Clinical characteristics, *n* (%)				
Acute coronary syndrome	107 (63.3)	65 (71.4)	42 (53.8)	0.018
Prior MI	23 (13.6)	13 (14.3)	10 (12.8)	0.782
Prior PCI	42 (24.9)	19 (20.9)	23 (29.5)	0.197
CCS 3/4 angina	130 (76.9)	75 (82.4)	55 (70.5)	0.067
NYHA 3/4	129 (76.3)	67 (73.6)	62 (79.5)	0.372
Comorbidities, *n* (%)				
Hypertension	128 (75.7)	68 (74.7)	60 (76.9)	0.740
Diabetes	61 (36.1)	30 (33.0)	31 (39.7)	0.360
Insulin-dependent	25 (14.8)	14 (15.4)	11 (14.1)	0.815
Hyperlipidemia	77 (45.6)	37 (40.7)	40 (51.3)	0.167
Smoking status				
Current	30 (17.8)	14 (15.4)	16 (20.5)	0.384
Never/former	139 (82.2)	77 (84.6)	62 (79.5)	
Atrial fibrillation	7 (4.1)	5 (5.5)	2 (2.6)	0.571
PVD	30 (17.8)	13 (14.3)	17 (21.8)	0.203
Renal insufficiency	29 (17.2)	11 (12.1)	18 (23.1)	0.059
COPD	18 (10.7)	6 (6.6)	12 (15.4)	0.065
Cerebrovascular disease	47 (27.8)	20 (22.0)	27 (34.6)	0.068
LVED (mm), median (Q1, Q3)	48.00 (46.00, 50.00)	48.00 (46.00, 51.00)	48.00 (46.00, 50.00)	0.784
LVEF (%), median (Q1, Q3)	64.00 (60.00, 68.00)	65.00 (62.00, 68.00)	63.00 (60.00, 67.00)	0.151
Hemoglobin (g/L), mean ± SD	133.01 ± 15.66	131.96 ± 15.26	134.24 ± 16.12	0.345
eGFR (mL/min/1.73 m^2^), median (Q1, Q3)	79.60 (67.20, 90.60)	83.60 (70.25, 91.60)	75.85 (59.88, 89.90)	0.100
hs-CRP (mg/L), median (Q1, Q3)	3.50 (1.00, 3.74)	3.70 (1.06, 3.94)	2.16 (0.82, 3.70)	0.251
Number of diseased coronary vessels, *n* (%)				0.068
Two	41 (24.3)	17 (18.7)	24 (30.8)	
Three	128 (75.7)	74 (81.3)	54 (69.2)	
Left main disease, *n* (%)	51 (30.2)	33 (36.3)	18 (23.1)	0.063
Mitral regurgitation, *n* (%)				0.699
None/mild	118 (69.8)	61 (67.0)	57 (73.1)	
Moderate	49 (29.0)	29 (31.9)	20 (25.6)	
Severe	2 (1.2)	1 (1.1)	1 (1.3)	
Aortic regurgitation, *n* (%)				0.296
None/mild	132 (78.1)	68 (74.7)	64 (82.1)	
Moderate	34 (20.1)	22 (24.2)	12 (15.4)	
Severe	3 (1.8)	1 (1.1)	2 (2.6)	

HCR, hybrid coronary revascularization; MICS-CABG, minimally invasive multivessel coronary artery bypass grafting; BMI, body mass index; MI, myocardial infarction; PCI, percutaneous coronary intervention; PVD, peripheral vascular disease; COPD, chronic obstructive pulmonary disease; LVED, left ventricular end-diastolic diameter; LVEF, left ventricular ejection fraction; eGFR, estimated glomerular filtration rate; hs-CRP, high-sensitivity C-reactive protein**.**

### Operative and early postoperative outcomes

Major surgical perioperative complications within 30 days after surgery were uncommon in both groups. In the unmatched cohort, MI occurred in 1 patient in the HCR group and in no patients in the MICS-CABG group (1.10% vs. 0.00%; *P* = 1.000), stroke occurred in 2 patients and no patients, respectively (2.20% vs. 0.00%; *P* = 0.500), and death occurred in 1 patient and no patients, respectively (1.10% vs. 0.00%; *P* = 1.000). After PSM, the results remained similar, with 1 MI (1.52% vs. 0.00%; *P* = 1.000), 2 strokes (3.03% vs. 0.00%; *P* = 0.476), and 1 death (1.52% vs. 0.00%; *P* = 1.000) in the HCR group, whereas no such events occurred in the MICS-CABG group.

In the unmatched cohort, operative time was significantly longer in the MICS-CABG group than in the HCR group (250 vs. 150 min, *P* < 0.001), with a greater number of surgical grafts (3 vs. 1, *P* < 0.001) and higher 24-hour postoperative drainage volume (430 vs. 165 mL, *P* < 0.001). Mechanical ventilation time also differed, with more HCR patients extubated within 12 h (*P* < 0.001). No significant differences were observed in perioperative transfusion, intensive care unit stay, postoperative length of stay, opioid analgesia, re-exploration for bleeding, respiratory complications, new-onset atrial fibrillation, or poor wound healing. After PSM, operative time remained longer in the MICS-CABG group (250 vs. 160 min, *P* < 0.001), with more grafts (3 vs. 1, *P* < 0.001) and higher 24-hour drainage (420 vs. 185 mL, *P* < 0.001). Mechanical ventilation time also continued to differ significantly, with 83.3% of patients in the HCR group extubated within 12 h compared with 36.4% in the MICS-CABG group (*P* < 0.001). Postoperative respiratory complications were numerically more frequent in the MICS-CABG group than in the HCR group, including acute respiratory distress syndrome (ARDS), HFNC use, and pulmonary infection, although none of these differences reached statistical significance (Table 2).

**Table 2:** Perioperative outcomes before and after propensity score matching.

Variable	Before PSM				After PSM			
	Total (*n* = 169)	HCR (*n* = 91)	MICS-CABG (*n* = 78)	*P*	Total (*n* = 132)	HCR (*n* = 66)	MICS-CABG (*n* = 66)	*P*
Operative time (min), median (Q1, Q3)	215.00 (150.00, 255.00)	150.00 (130.00, 180.00)	250.00 (235.00, 278.75)	<0.001	230.00 (160.00, 256.25)	160.00 (135.00, 180.00)	250.00 (235.00, 280.00)	<0.001
Transfusion, *n* (%)	16 (9.47)	7 (7.69)	9 (11.54)	0.395	11 (8.33)	3 (4.55)	8 (12.12)	0.115
Number of grafts, median (Q1, Q3)	2.00 (1.00, 3.00)	1.00 (1.00, 1.00)	3.00 (2.00, 3.00)	<0.001	2.00 (1.00, 3.00)	1.00 (1.00, 1.00)	3.00 (2.00, 3.00)	<0.001
24 h postoperative drainage (mL), median (Q1, Q3)	290.00 (155.00, 445.00)	165.00 (120.00, 232.50)	430.00 (370.00, 527.50)	<0.001	300.00 (182.50, 437.50)	185.00 (120.00, 253.75)	420.00 (365.00, 498.75)	<0.001
Mechanical ventilation time, *n* (%)				<0.001				<0.001
<12 h	98 (57.99)	73 (80.22)	25 (32.05)		79 (59.85)	55 (83.33)	24 (36.36)	
12–24 h	62 (36.69)	15 (16.48)	47 (60.26)		46 (34.85)	9 (13.64)	37 (56.06)	
>24 h	9 (5.33)	3 (3.30)	6 (7.69)		7 (5.30)	2 (3.03)	5 (7.58)	
ICU stay, *n* (%)				0.503				0.156
<48 h	117 (69.23)	61 (67.03)	56 (71.79)		95 (71.97)	43 (65.15)	52 (78.79)	
48–72 h	39 (23.08)	24 (26.37)	15 (19.23)		29 (21.97)	19 (28.79)	10 (15.15)	
>72 h	13 (7.69)	6 (6.59)	7 (8.97)		8 (6.06)	4 (6.06)	4 (6.06)	
Postoperative hospital stay(d), median (Q1, Q3)	7.00 (6.00, 9.00)	8.00 (6.00, 9.00)	7.00 (7.00, 9.00)	0.932	7.00 (6.00, 9.00)	7.00 (6.00, 9.00)	7.00 (7.00, 9.00)	0.536
Conversion to sternotomy, *n* (%)	-	-	-	-	-	-	-	-
IABP support, *n* (%)	-	-	-	-	-	-	-	-
ECMO support, *n* (%)	-	-	-	-	-	-	-	-
Opioid analgesia, *n* (%)	17 (10.06)	8 (8.79)	9 (11.54)	0.554	13 (9.85)	6 (9.09)	7 (10.61)	0.770
Re-thoracotomy for bleeding, *n* (%)	1 (0.59)	1 (1.10)	0 (0.00)	1.000	1 (0.76)	1 (1.52)	0 (0.00)	1.000
Major complications within 30 days, *n* (%)								
Myocardial infarction	1 (0.59)	1 (1.10)	0 (0.00)	1.000	1 (0.76)	1 (1.52)	0 (0.00)	1.000
Stroke	2 (1.18)	2 (2.20)	0 (0.00)	0.500	2 (1.52)	2 (3.03)	0 (0.00)	0.476
Death	1 (0.59)	1 (1.10)	0 (0.00)	1.000	1 (0.76)	1 (1.52)	0 (0.00)	1.000
Respiratory complications, *n* (%)								
ARDS	29 (17.16)	12 (13.19)	17 (21.79)	0.139	26 (19.70)	11 (16.67)	15 (22.73)	0.381
Reintubation	3 (1.78)	1 (1.10)	2 (2.56)	0.893	2 (1.52)	1 (1.52)	1 (1.52)	1.000
HFNC, *n* (%)	30 (17.75)	12 (13.19)	18 (23.08)	0.093	26 (19.70)	11 (16.67)	15 (22.73)	0.381
Pulmonary infection, *n* (%)	7 (4.14)	2 (2.20)	5 (6.41)	0.326	3 (2.27)	1 (1.52)	2 (3.03)	1.000
New-onset atrial fibrillation, *n* (%)	10 (5.92)	4 (4.40)	6 (7.69)	0.563	3 (2.27)	1 (1.52)	2 (3.03)	1.000
Poor wound healing, *n* (%)	11 (6.51)	6 (6.59)	5 (6.41)	0.962	8 (6.06)	4 (6.06)	4 (6.06)	1.000

HCR, hybrid coronary revascularization; MICS-CABG, minimally invasive multivessel coronary artery bypass grafting; PSM, propensity score matching; ICU, intensive care unit; ARDS, acute respiratory distress syndrome; HFNC, high-flow nasal cannula; IABP, intra-aortic balloon pump; ECMO, extracorporeal membrane oxygenation.

### Mid-term clinical outcomes

All-cause death occurred in one patient in the HCR group and one patient in the MICS-CABG group. In the HCR group, one patient died in hospital within 30 days after surgery, after completion of staged PCI, due to heart failure complicated by multiple organ failure. In the MICS-CABG group, one patient died after hospital discharge, more than 30 days after surgery, due to heart failure. Before PSM, the 1-year survival rate was 98.9% in the HCR group and 98.7% in the MICS-CABG group, with no significant difference between the two groups (log-rank *P* = 0.918) (Figure 2A).

**Figure 2:**
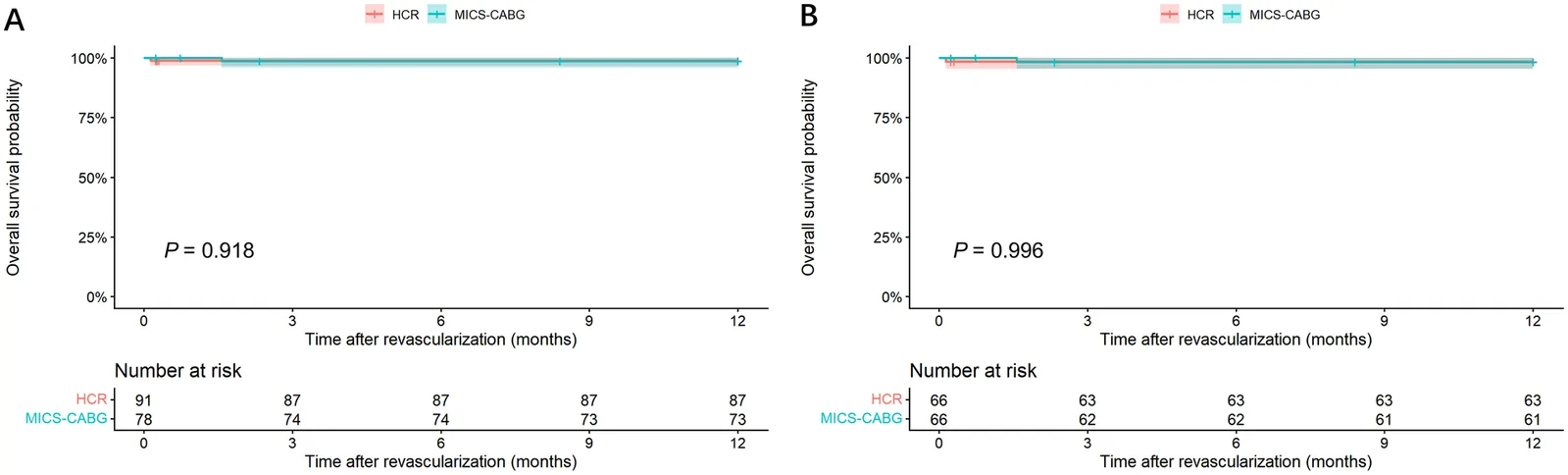
Kaplan–Meier survival curves comparing HCR and MICS-CABG groups. **(A)** Survival curves in the cohort before PSM. **(B)** Survival curves in the cohort after PSM.

After PSM, all-cause death occurred in one patient in each group. The 1-year survival rate after matching was 98.5% in the HCR group and 98.4% in the MICS-CABG group, with no significant between-group difference (log-rank *P* = 0.996) (Figure 2B).

### One-year revascularization patency

In the MICS-CABG group, 52 of 78 patients (66.7%) underwent follow-up imaging, including 49 patients assessed by CCTA and 3 by CAG. In the HCR group, follow-up imaging was completed in 57 of 91 patients (62.6%), including 52 CCTA and 5 CAG assessments.

LAD revascularization was individualized according to coronary anatomy and target-vessel characteristics. In the HCR group, most patients underwent standard LIMA-to-LAD anastomosis, whereas alternative strategies included one sequential LIMA–SVG–diagonal branch–LAD graft, two SVG-to-LAD grafts, and PCI of a small LAD vessel in one patient. In the MICS-CABG group, two patients did not undergo LAD grafting because of insufficient surgical indication. Alternative LAD graft configurations included two sequential LIMA–diagonal branch–LAD grafts, one LIMA–SVG–LAD graft, three RIMA-to-LAD grafts, and one SVG-to-LAD graft.

Overall, among the 57 HCR patients who underwent follow-up imaging, 56 LAD grafts were available for graft patency assessment; the remaining patient had undergone PCI rather than bypass grafting for a small LAD and was therefore not included in the LAD graft patency analysis. In the MICS-CABG group, 50 LAD grafts were available for graft patency assessment. The combined FitzGibbon A + B patency rate was 94.6% in the HCR group and 90.0% in the MICS-CABG group, with no significant between-group difference (*χ*^2^ = 0.816, *P* = 0.366). For non-LAD target vessels, 89 vessels were evaluated in the HCR group and 100 in the MICS-CABG group, with comparable patency rates between groups (86.5% vs. 87.0%; *χ*^2^ = 0.010, *P* = 0.922) (Figure 3).

**Figure 3:**
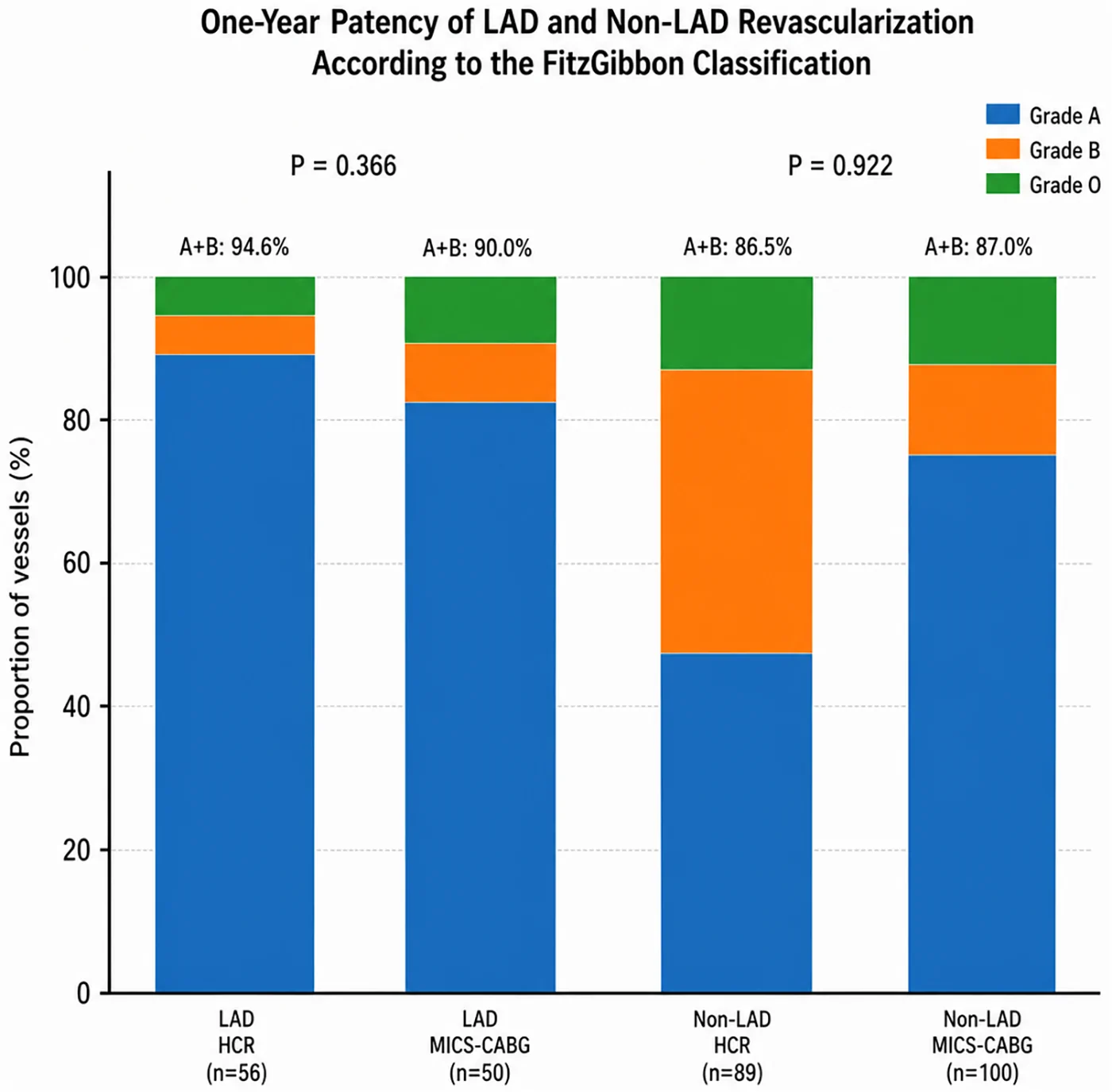
One-year patency of LAD and non-LAD revascularization according to the FitzGibbon classification.

## Discussion

In this single-center retrospective cohort study, both HCR and MICS-CABG showed favorable perioperative safety and comparable early-to-mid-term outcomes in patients with MVCAD. After propensity score matching, there were no significant differences between groups in major perioperative adverse events, 1-year survival, or 1-year LAD and non-LAD target-vessel patency. Compared with HCR, MICS-CABG was associated with a significantly longer operative time, greater 24-hour postoperative drainage volume, and delayed early extubation. These differences may be attributable to the technical complexity of MICS-CABG, which requires complete surgical revascularization through a limited thoracotomy, involving additional conduit harvesting, multiple distal and proximal anastomoses, and prolonged cardiac manipulation.

Despite the longer operative time and greater postoperative drainage, these differences did not translate into significantly longer ICU or postoperative hospital stays or higher rates of transfusion or re-exploration for bleeding. However, delayed early extubation and numerically more frequent respiratory complications may indicate a greater early postoperative recovery burden after MICS-CABG. The potential impact on cost and patient-reported recovery was not evaluated in the present study and warrants further investigation.

Although the differences in respiratory complications did not reach statistical significance, possibly owing to the limited sample size, the MICS-CABG group showed a numerically greater postoperative pulmonary burden, including higher incidences of ARDS and HFNC use, whereas reintubation rates were comparable after PSM. Prolonged one-lung ventilation may impair postoperative pulmonary function, and re-expansion of the collapsed lung after restoration of bilateral ventilation may contribute to ischemia–reperfusion injury, inflammatory cytokine release, atelectasis, and ventilation–perfusion mismatch (8). In addition, more extensive intrathoracic manipulation during multivessel surgical revascularization may increase the risk of pleural effusion, hemothorax, pericardial effusion, and postoperative atelectasis, thereby contributing to delayed respiratory recovery (9). Therefore, these findings suggest that perioperative pulmonary-protective strategies may be important in MICS-CABG. Notably, pulmonary-protective measures were introduced progressively during the study period rather than uniformly applied to all MICS-CABG patients, which may have influenced the observed incidence of respiratory complications. Potential measures include shortening the duration of one-lung ventilation, restoring bilateral ventilation as early as feasible, applying apneic oxygen insufflation to the non-ventilated lung when appropriate, and minimizing excessive cardiac compression while maintaining adequate surgical exposure (10). In addition, optimized postoperative analgesia, smoking cessation, and perioperative respiratory training may help promote postoperative pulmonary recovery, particularly in patients with limited cardiopulmonary reserve (11).

The similar 1-year survival observed between the HCR and MICS-CABG groups suggests that, when applied to carefully selected patients, both minimally invasive strategies may provide comparable early-to-mid-term survival outcomes despite their different revascularization mechanisms. The comparable graft- and target-vessel patency observed in our study may partly explain these similar clinical outcomes. In both strategies, revascularization of the LAD territory was predominantly based on the LIMA–LAD graft, which is widely recognized as the most durable component of coronary revascularization (12, 13). However, follow-up imaging was available in only 62.6% of HCR patients and 66.7% of MICS-CABG patients. Although the follow-up rates were similar between groups, selection bias cannot be excluded, as patients who underwent imaging may not fully represent the entire cohort. Therefore, the observed patency rates and between-group comparisons should be interpreted cautiously.

For non-LAD territories, however, the mechanisms of durability differ: HCR relies on drug-eluting stents, whereas MICS-CABG depends on bypass grafts, including arterial or venous conduits. Therefore, the similar non-LAD target-vessel patency observed in our cohort suggests that, in carefully selected patients, PCI-based completion of revascularization in HCR and graft-based complete surgical revascularization in MICS-CABG may achieve comparable short-term anatomical efficacy. Nevertheless, because stent failure and graft failure are driven by different mechanisms, longer follow-up is required to determine whether these two strategies remain comparable in terms of late target-vessel failure, repeat revascularization, and graft or stent durability.

The 30-day perioperative window was counted from the date of minimally invasive surgery to evaluate surgical perioperative safety. However, this definition may not fully capture the cumulative risk of the staged HCR strategy. In particular, adverse events could theoretically occur while HCR patients were awaiting staged PCI. To address this issue, we carefully reviewed all HCR cases and confirmed that no all-cause death, MI, or stroke occurred during the interval. Nevertheless, future studies should report both surgery-based and strategy-based 30-day outcomes to more comprehensively evaluate the overall safety of HCR. Robotic assistance may facilitate internal mammary artery harvesting during minimally invasive coronary surgery. In the present cohort, robotic assistance was used for internal mammary artery harvesting in only two HCR patients, while no MICS-CABG patients underwent robotic procedures. Given the very small number of robotic cases, meaningful subgroup analysis was not feasible. Further studies are needed to evaluate the potential role of robotic techniques, particularly for bilateral internal mammary artery harvesting in MICS-CABG. This study has several limitations, including its single-center retrospective design, modest sample size, and limited number of long-term events, which may reduce statistical power and introduce selection bias. In addition, imaging follow-up was incomplete, with approximately two-thirds of patients undergoing patency assessment at 1 year. This may have introduced selection bias, because patients who returned for CCTA or CAG may have differed from those without imaging follow-up in clinical symptoms, adherence to follow-up or clinical risk. Nevertheless, the imaging follow-up rates were broadly similar between the HCR and MICS-CABG groups. Larger prospective multicenter studies with longer follow-up are warranted to confirm these findings and determine the optimal minimally invasive revascularization strategy for different patient subgroups.

## Conclusions

In this single-center retrospective cohort of selected patients with multivessel coronary artery disease, no statistically significant differences were observed between HCR and MICS-CABG in 30-day major adverse events, 1-year survival, or 1-year LAD and non-LAD target-vessel patency after PSM. MICS-CABG was associated with longer operative time, greater early postoperative drainage, and delayed extubation. These findings require validation in larger prospective studies with longer follow-up.

## Data Availability

The raw data supporting the conclusions of this article will be made available by the authors, without undue reservation.

## Supplementary material

Supplementary Figure 1 Covariate balance before and after propensity score matching. (A) Absolute standardized mean differences for key baseline covariates.(B) Distribution of propensity scores in the treatment group (HCR, 1) and control group (MICS-CABG, 0) before and after matching.
